# Neurorehabilitation and Functional Improvement in Joubert Syndrome: A 12-Month Case Report

**DOI:** 10.3390/children13040452

**Published:** 2026-03-26

**Authors:** Łukasz Mański, Aleksandra Moluszys, Eliza Wasilewska, Agnieszka Rosa, Krzysztof Szczałuba, Jan Szumlicki, Krystyna Szymańska, Jolanta Wierzba

**Affiliations:** 1 Gdansk Medical Academy of Applied Sciences, 80-335 Gdansk, Poland; aleksandra.moluszys@gam.edu.pl; 2 Department of Allergology, Medical University of Gdansk, 80-210 Gdansk, Poland; 3 Center of Excellence for Rare and Undiagnosed Disorders, Medical University of Warsaw, 02-091 Warsaw, Poland; agarosa@vp.pl (A.R.); krzysztof.szczaluba@wum.edu.pl (K.S.);; 4 Department of Pediatric Neurology and Rare Disorders, Medical University of Warsaw, 02-091 Warsaw, Poland; 5 Department of Internal and Pediatric Nursing, Institute of Nursing and Midwifery, Medical University Gdansk, 80-208 Gdansk, Poland

**Keywords:** Joubert syndrome, neurorehabilitation, Vojta therapy, postural control, pediatric rehabilitation, respiratory-postural integration, case report

## Abstract

**Highlights:**

**What are the main findings?**

- A 12-month mechanism-oriented neurorehabilitation programme was associated with substantial improvement in gross motor function in a child with Joubert syndrome (GMFM-88: 12% to 52%).
- Multidimensional assessment demonstrated concurrent improvements in respiratory-postural integration and functional visual performance, indicating enhanced sensorimotor organization.

**What are the implications of the main findings?**

- Mechanism-oriented neurorehabilitation targeting axial stabilization and respiratory-postural coupling may positively influence functional trajectories in cerebellar and brainstem disorders.
- Multidimensional outcome tracking, including visual, respiratory, and postural domains, may improve sensitivity to rehabilitation-related changes beyond motor scales alone.

**Abstract:**

Background: Joubert syndrome (JS) is a rare ciliopathy characterized by cerebellar and brainstem malformations and the molar tooth sign on magnetic resonance imaging. Motor impairment is primarily driven by axial hypotonia, impaired postural control, and disrupted respiratory-postural integration. Longitudinal reports describing structured neurorehabilitation with standardized functional outcomes remain limited. Case presentation: We report a female child with prenatally suspected vermian hypoplasia and postnatally MRI-confirmed Joubert syndrome. Subsequent molecular testing performed at the age of 3 years and 11 months identified heterozygous variants in the *B9D2* gene associated with Joubert syndrome. Early development was marked by axial hypotonia, global motor delay, impaired trunk stabilization, sleep-disordered breathing, and early hip migration. At 2.5 years of age, following motor plateau under conventional therapy, a structured 12-month rehabilitation programme was introduced, combining Vojta-based reflex locomotion, respiratory therapy targeting thoraco-diaphragmatic synchronization, daily home-based practice, and supported standing. Results: After 12 months, gross motor function improved substantially, with GMFM-88 increasing from 12% to 52% (+40 percentage points). PEDI scaled scores improved across all domains, with mobility increasing from 8 to 40, self-care from 15 to 45, and social function from 25 to 50. Ataxia severity decreased from 22 to 15 on the modified Brief Ataxia Rating Scale, consistent with improved trunk stability and coordination. Postural and respiratory organization improved, reflected by a reduction in the subcostal angle from 137° to 90°, an increase in sacral slope from 5° to 10°, and increased expiratory pressure from 10 to 25 mmHg. Caregiver-reported assessment combined with structured clinical observation indicated improved functional visual performance, including enhanced visual attention, visuomotor coordination, and environmental visual interaction. Conclusions: Structured neurorehabilitation was associated with substantial functional improvement across motor, postural, and respiratory domains. These findings support the clinical relevance of mechanism-oriented neurorehabilitation and standardized longitudinal outcome assessment in Joubert syndrome.

## 1. Introduction

Joubert syndrome (JS) is a rare congenital ciliopathy characterized by malformations of the cerebellum and brainstem. The radiological hallmark of the disorder is the molar tooth sign on magnetic resonance imaging (MRI), reflecting vermian hypoplasia and abnormal configuration of the superior cerebellar peduncles. The estimated prevalence of JS ranges from approximately 1 in 80,000 to 1 in 100,000 live births [1], although the true prevalence may be underestimated due to diagnostic challenges and phenotypic variability [2,3]. Genetically, JS belongs to a group of disorders associated with dysfunction of the primary cilium. Pathogenic variants have been identified in more than 40 genes involved in ciliary structure and signaling pathways [4].

Clinically, JS presents with axial hypotonia, ataxia, abnormal ocular movements, respiratory dysregulation, and variable developmental delay. Multisystem involvement is common and may include retinal dystrophy, renal disease, hepatic fibrosis, and skeletal abnormalities, contributing to substantial phenotypic heterogeneity [2]. These features reflect dysfunction of cerebellar and brainstem structures responsible for the integration of postural, respiratory, and sensorimotor control [5]. Disruption of these networks interferes with the regulation of muscle tone, postural alignment, and coordination of voluntary movements. Consequently, children with JS frequently demonstrate impaired trunk stability, delayed acquisition of gross motor milestones, and persistent limitations in functional mobility. Respiratory irregularities and oculomotor disturbances may further affect motor organization and participation, highlighting the central role of cerebellar-brainstem circuits in the development of coordinated motor behavior [6].

Although early rehabilitation is widely recognized as a critical component of management in JS, evidence guiding specific physiotherapeutic strategies remains limited due to the rarity of the condition, phenotypic heterogeneity, and the scarcity of longitudinal rehabilitation studies. Current knowledge regarding rehabilitation in this population is largely derived from small case series or isolated clinical observations, and standardized therapeutic frameworks have not yet been established. Most published reports focus primarily on diagnostic features, genetic findings, or short-term developmental descriptions rather than structured rehabilitation approaches. Consequently, detailed longitudinal descriptions of physiotherapy programmes including therapeutic rationale, intervention parameters, and multidimensional functional outcomes remain limited in the current literature [7,8,9].

Among physiotherapeutic approaches used in children with central hypotonia and neurodevelopmental disorders, the Vojta method (reflex locomotion therapy) aims to activate innate postural and locomotor patterns through stimulation of specific peripheral zones [10]. This stimulation is thought to engage subcortical motor circuits, including brainstem and spinal pathways, thereby facilitating postural activation and sensorimotor integration. Although evidence remains limited and heterogeneous and is largely derived from studies conducted in other pediatric populations with motor impairment, this approach has been explored in children presenting with impaired postural control and central hypotonia [11,12].

The aim of this case report was to describe the clinical course and functional outcomes of a child with JS undergoing a structured 12-month mechanism-oriented neurorehabilitation programme targeting axial stabilization and respiratory-postural integration. We sought to evaluate longitudinal changes in gross motor function, functional independence, and postural-respiratory organization using standardized multidimensional outcome measures. This report may serve as an illustrative example of mechanism-oriented neurorehabilitation in cerebellar and brainstem disorders.

## 2. Case

### 2.1. Perinatal History

The patient was delivered via elective caesarean section at 39 + 2 weeks of gestation. The procedure was uncomplicated. The neonate required no resuscitation and was clinically stable. The Apgar score was 10 at 1 min. Muscle tone and spontaneous respiration were normal. Birth measurements were within normal percentiles: weight 3170 g, length 53 cm, head circumference 34 cm, and chest circumference 33 cm. She was born at term and classified as appropriate for gestational age. She was the second child of healthy, non-consanguineous parents. There was no family history of genetic, neurological, or developmental disorders. Written informed consent was obtained from the patient’s legal guardians for publication of this case report and accompanying images.

### 2.2. Prenatal and Postnatal Findings

A routine third-trimester ultrasound raised suspicion of a posterior fossa anomaly. The fourth ventricle appeared enlarged, and the cerebellar vermis appeared underdeveloped. Fetal MRI was not performed. Postnatal transfontanelle ultrasound confirmed posterior fossa abnormalities.

At 10 months of age, brain MRI revealed agenesis of the cerebellar vermis, thickened and elongated superior cerebellar peduncles, enlargement of the fourth ventricle with a batwing configuration, and a deepened interpeduncular fossa. These findings formed the molar tooth sign and were consistent with JS.

Subsequent molecular testing performed at the age of 3 years and 11 months identified two variants in the *B9D2* gene, associated with JS and related ciliopathies, were identified: c.513C>G located in exon 4 and c.146C>T located in exon 3. The latter variant is currently classified as a variant of uncertain significance. The identification of these variants provided molecular support for the clinical and radiological diagnosis.

### 2.3. Clinical Course

The neonatal period was marked by pronounced axial hypotonia, weak cry, and ineffective feeding. Oral intake was delayed and rooting and sucking reflexes were diminished. During the first months of life, global developmental delay became evident. Axial hypotonia persisted and impaired head control. At six months, head lag was present during pull-to-sit, and spontaneous antigravity movements were limited. The Landau reaction was weak and poorly sustained, consistent with reduced axial extensor activation. The child was unable to roll or initiate midline reaching, and postural control in the prone position was markedly reduced.

Sleep-wake dysregulation appeared by the third month of life, with frequent nocturnal awakenings and irregular sleep patterns. Oculomotor examination revealed intermittent strabismus and rotatory nystagmus. Mild oromotor dysfunction resulted in prolonged feeding and occasional choking. By the end of the first year, recurrent upper respiratory tract infections, including adenoid hypertrophy and pharyngitis, were documented and required otolaryngology follow-up. Neurological assessment confirmed persistent axial hypotonia, motor incoordination, and delayed acquisition of postural and locomotor milestones.

## 3. Diagnostic Assessment

**Figure 1:**
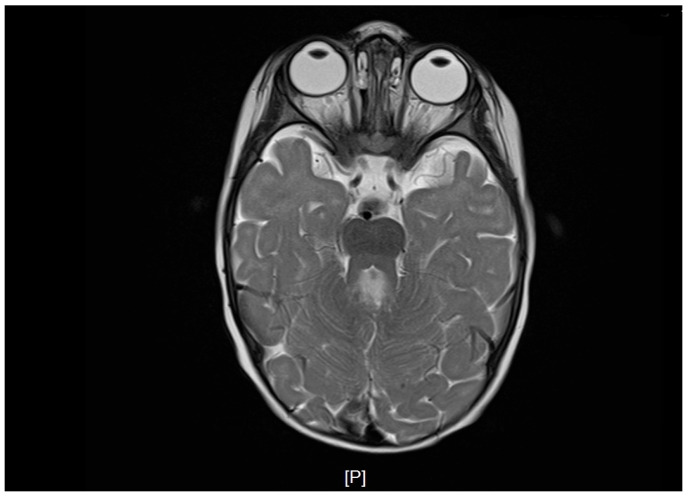
Brain MRI demonstrating the molar tooth sign characteristic of Joubert syndrome. Axial T2-weighted image shows vermian agenesis and elongated superior cerebellar peduncles.

**Figure 2:**
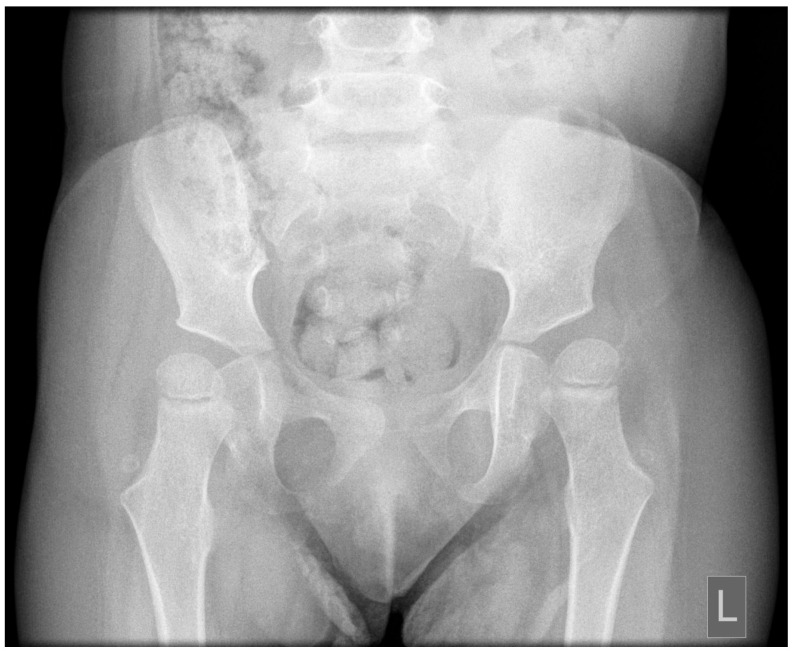
Pelvic radiograph demonstrating asymmetric lateral migration of the femoral heads, more pronounced on the left side, consistent with early hip displacement.

Postnatal brain MRI confirmed the diagnosis of JS based on the presence of the molar tooth sign (Figure 1). Polysomnography performed at 1 year of age demonstrated prolonged sleep latency (112 min), moderate mixed sleep apnea (AHI 5.4/h), and significant oxygen desaturation with a nadir SpO_2_ of 77%. Ophthalmologic follow-up confirmed persistent bilateral hyperopia with astigmatism requiring optical correction, consistent with cerebellar-related visual instability. Pelvic radiography performed at 2.5 years of age revealed asymmetric lateral migration of the femoral heads, more pronounced on the left side, with reduced acetabular coverage consistent with early subluxation (Figure 2). Clinically, the left lower limb presented with persistent adduction positioning. Diagnostic findings demonstrated cerebellar and brainstem abnormalities with direct functional implications for postural control, respiratory regulation, and motor development (Table 1).

The Gross Motor Function Measure (GMFM-88) was originally developed and validated for children with cerebral palsy and has not been specifically validated for Joubert syndrome. However, it has also been applied in a range of pediatric conditions with motor impairment including acquired brain injury, and neuromuscular disorders, where studies have demonstrated its reliability and clinical utility for assessing gross motor function and monitoring longitudinal functional changes. In the present case, GMFM-88 was therefore used as a structured tool to track changes in gross motor performance over time [13,14]. In addition to GMFM-88, physiotherapeutic functional assessment included the Pediatric Evaluation of Disability Inventory (PEDI) to evaluate functional independence and participation, the modified Brief Ataxia Rating Scale (mBARS) to characterize cerebellar-related motor impairment and selected postural-respiratory parameters including the subcostal angle and sacral slope to assess respiratory–postural integration. These parameters were used as descriptive clinical indicators of postural-respiratory organization, as standardized pediatric reference values for these measurements are currently lacking. Expiratory pressure was assessed using a Positive Expiratory Pressure System-S (PARI PEP System-S; PARI GmbH, Starnberg, Germany) device connected to a manometer at a standardized resistance level of 1.5, with the child positioned in the supine position to minimize compensatory trunk activation and ensure measurement consistency. Measurements were performed by an experienced pediatric respiratory physiotherapist, and the highest stable value was recorded for analysis. The measurement was therefore used as a clinical indicator of expiratory pressure generation rather than as a standardized physiological outcome measure. Functional visual performance was additionally assessed using a structured caregiver-reported questionnaire combined with standardized clinical observation performed at baseline and follow-up.

The patient was enrolled in a multidisciplinary care pathway including pediatric neurology, physiotherapy, respiratory therapy, speech and oromotor therapy, vision therapy, otolaryngology, pulmonology, orthopedics, and psychological services.

## 4. Therapeutic Intervention

Until the age of 2.5 years, the rehabilitation programme consisted of conventional neurodevelopmental therapy, visual stimulation, and basic oromotor facilitation supported by a structured home routine. Despite consistent implementation, the rate of motor progress appeared to slow over time. At the age of 2.5 years, in response to the observed plateau in motor development, a revised functionally oriented rehabilitation protocol was introduced and continued for 12 months.

In the present report, the term mechanism-oriented neurorehabilitation refers to a therapeutic strategy targeting specific physiological mechanisms underlying motor impairment, particularly axial stabilization, respiratory-postural coupling, and sensorimotor integration. In contrast to conventional rehabilitation approaches primarily focused on practicing developmental motor tasks or milestones, this approach emphasizes activation of underlying regulatory mechanisms that support postural control and coordinated movement.

The programme included Vojta-based reflex locomotion therapy delivered once weekly by a certified pediatric physiotherapist with 8 years of clinical experience. Each session lasted approximately 30–40 min depending on the child’s clinical condition and tolerance. The intervention consisted primarily of reflex creeping and reflex rolling patterns with stimulation of standard Vojta activation zones. Stimulation was applied in repeated activation cycles separated by short rest periods and adjusted according to therapeutic goals. Emphasis was placed on facilitation of axial stabilization and postural activation. This approach was selected to promote activation of subcortical motor circuits involved in postural control, including brainstem-mediated pathways that contribute to the organization of locomotor and postural functions during development.

At the beginning of each session, the therapist discussed the child’s current condition with the caregivers and performed brief clinical observation of spontaneous movement, postural control, and overall motor coordination. Tolerance to therapy was monitored continuously throughout the intervention based on changes in movement quality, coordination, behavioural responses, and signs of fatigue. Simple goal-directed activities, such as reaching toward a toy, were occasionally used as practical indicators of movement organization and coordination. If deterioration in movement quality, increasing incoordination, or clear signs of fatigue were observed, the session was paused or terminated to avoid reinforcing compensatory movement patterns and to maintain optimal motor learning conditions.

A structured home-based programme was implemented under caregiver supervision. Parents (mother and father), previously trained by the therapists and supported by video demonstrations provided during therapy sessions, performed Vojta stimulation once to twice daily depending on the child’s condition and daily schedule. Each home session lasted approximately 10 min and was followed by play-based activities encouraging spontaneous use of developmentally appropriate motor patterns available to the child at that stage of development, aimed at reinforcing functional motor organization. Adherence to the home programme was monitored through regular caregiver feedback and observation during follow-up visits. Caregivers were also encouraged to record short videos of home exercises, which were reviewed during subsequent therapy sessions to provide feedback and verify correct performance.

Respiratory therapy was provided by an experienced respiratory physiotherapist (10 years of clinical experience) approximately every two weeks or monthly depending on the child’s clinical status and therapist availability. The intervention focused on respiratory re-education and respiratory-postural integration, including diaphragmatic breathing facilitation, manual mobilization of intercostal spaces, activation of accessory respiratory muscles, and positive expiratory pressure training using a PEP device. Caregivers additionally received home-based recommendations following each visit. In cases of respiratory infections such as bronchitis or pneumonia, respiratory therapy sessions were temporarily postponed until recovery. The intensity of the intervention was temporarily reduced following illnesses when the child demonstrated increased fatigue or reduced movement quality.

Due to documented hip migration, a supported standing programme was introduced using a standing frame. During the first 2–3 weeks, the child was progressively accustomed to verticalization. Subsequently, supported standing was performed once daily for approximately 45–60 min depending on tolerance. Hip abduction was set at approximately 25° to promote symmetrical loading of the hip joints and improve femoral head positioning, thereby reducing the risk of progressive hip displacement associated with axial hypotonia. During standing sessions, the child additionally used ankle-foot orthoses (AFOs) to improve lower limb alignment and provide stable support during verticalization.

Speech and language therapy was provided by a speech and language therapist specializing in neurological disorders, responsible for augmentative and alternative communication (AAC) training and oromotor interventions. Caregivers also received home-based recommendations and exercises supporting communication. Visual therapy sessions were conducted by a vision therapist; however, due to limited availability of specialists, these sessions were performed approximately once per month or less frequently depending on service accessibility.

Clinical progress was monitored regularly during follow-up visits. Approximately once per month, functional reassessment was performed, including evaluation of joint range of motion, postural alignment, and selected clinical indicators of respiratory-postural organization. These observations were used to adjust the rehabilitation programme and guide therapeutic progression.

Temporary interruptions in the therapy schedule occasionally occurred due to intercurrent illness or family circumstances.

The primary therapeutic goals included improvement of axial stabilization, facilitation of functional motor patterns, and prevention of secondary musculoskeletal complications such as hip displacement.

## 5. Functional Outcomes and Follow-Up

After 12 months of intervention, quantitative changes were observed across multiple standardized outcome measures (Table 2). GMFM-88 increased from 12% at baseline (2.5 years of age) to 52% after 12 months of intervention (3.5 years of age), with the greatest gains in dimensions A–C. The magnitude of change (40 percentage points) substantially exceeds previously reported minimal clinically important differences for gross motor function measures in children with motor impairment [15]. PEDI scaled scores improved substantially across all functional domains. Mobility increased from 8 at baseline to 40 after 12 months, self-care from 15 to 45, and social function from 25 to 50, indicating marked gains in functional independence and participation. Ataxia severity was assessed using a mBARS; range 0–30), adapted descriptively for pediatric use to better capture axial and postural impairments relevant to early motor development. This adapted version was used to characterize longitudinal clinical change and was not intended as a formally validated outcome measure. The score improved from 22 at baseline to 15 after 12 months, indicating a 7-point reduction (32% relative change) consistent with clinically meaningful improvement in trunk stability and postural coordination.

The subcostal angle, measured twice with a manual goniometer in the supine position by an experienced pediatric physiotherapist and averaged for analysis, decreased from approximately 137° to 90°, suggesting improved thoraco-diaphragmatic organization. Sacral slope, assessed twice using a goniometer in the prone position by the same examiner and averaged for consistency, increased from 5° to 10°, indicating improved sagittal pelvic alignment. The observed increase in expiratory pressure from 10 mmHg to 25 mmHg may reflect improved expiratory pressure generation capacity, potentially consistent with enhanced respiratory-postural interaction.

At 3.5 years of age, following 12 months of structured neurorehabilitation, the child was able to transition independently into a seated position, initiate short crawling sequences, and participate in supported standing. Independent ambulation was not achieved. Improved trunk stability and postural symmetry were clinically evident. Feeding efficiency improved, with better lip closure and reduced drooling, allowing progression to semi-solid and soft solid textures. According to parental reports, sleep quality improved during the follow-up period, with fewer nocturnal awakenings and more regular sleep patterns. Caregiver-reported visual function assessment performed at 1 year of age and repeated at 3.5 years demonstrated improvement across multiple domains, including visual attention, visuomotor coordination, object recognition, and environmental visual interaction. The child showed increased visual engagement and more effective interaction with the environment, consistent with improved sensorimotor integration and functional visual performance (Table 3).

## 6. Discussion

This case report describes longitudinal functional changes observed during a structured neurorehabilitation programme targeting axial control and respiratory-postural integration in a child with JS. While causal inference cannot be established in a single-case design, the temporal relationship between the rehabilitation programme and multidimensional functional changes highlights the potential relevance of brainstem-mediated postural and respiratory mechanisms in rehabilitation strategies for cerebellar and brainstem disorders. In a disorder characterized by cerebellar vermian agenesis and brainstem dysfunction, motor impairment reflects disrupted axial tone regulation, impaired intersegmental coordination, and altered integration of vestibular, visual, and proprioceptive inputs essential for postural control [16,17,18]. The weak and poorly sustained Landau reaction observed early in life indicated deficient axial extensor recruitment, consistent with dysfunction of brainstem-mediated postural control circuits, particularly involving reticulospinal pathways responsible for axial muscle activation and postural stabilization [19,20]. The initial plateau under conventional neurodevelopmental therapy may therefore have been related to insufficient engagement of automatic postural control mechanisms. In the revised rehabilitation programme, reflex-based stimulation was complemented by play-based activities performed at home, encouraging spontaneous use of developmentally appropriate motor patterns during everyday interactions.

The rationale for introducing Vojta therapy was based on neurophysiological considerations related to subcortical motor control. Stimulation of defined peripheral zones is thought to generate afferent input projecting to brainstem nuclei and cerebellar structures, potentially engaging reticulospinal and vestibulospinal pathways and facilitating integration within cerebellar-brainstem motor networks [21,22,23]. In the context of impaired cortical-subcortical integration, repeated activation of these reflex-based postural programs may support recruitment of innate postural synergies independently of volitional motor planning [24,25]. However, evidence supporting the effectiveness of Vojta therapy specifically in JS remains limited, and the proposed mechanisms are largely extrapolated from studies conducted in other populations, including healthy subjects and children with neurological conditions such as Down syndrome or central hypotonia. Systematic reviews of rehabilitation interventions in pediatric neurological conditions have also highlighted the limited and inconclusive evidence supporting Vojta-based approaches for improving motor function [26]. Therefore, the present observations should be interpreted cautiously within the context of a single-case report. The observed improvement in GMFM dimensions A-C, together with the reduction in mBARS score, is consistent with improved axial stabilization. This pattern may precede distal motor progression through enhanced automatic postural control.

Although evidence regarding rehabilitation strategies in JS remains limited, several case reports and small case series have described physiotherapeutic and multidisciplinary rehabilitation approaches in this population [7,8,9,27,28,29,30,31,32]. Reported interventions include neurodevelopmental therapy based on the Bobath concept [7,8], play-based physiotherapy combined with sensory integration strategies [31], dynamic neuromuscular stabilization (DNS) targeting core stability [32], and multidisciplinary rehabilitation programs involving physiotherapy, occupational therapy, speech therapy, and orthotic management [9,27,28,30]. These interventions generally aim to improve trunk stability, postural control, motor milestone acquisition, and functional independence. Outcome measures reported in previous studies have included developmental scales, functional motor assessments such as GMFM-88 and Wee Functional Independence Measure (WeeFIM), and adaptive behavior scales including Vineland Adaptive Behavior Scales, Second Edition (Vineland-II) [8,28,29,30,31,32]. Across these reports, gradual improvements in motor function, trunk control, and functional independence have been described, although the heterogeneity of interventions and outcome measures limits direct comparison between studies.

Concurrent respiratory intervention targeted thoraco-diaphragmatic coordination and intra-abdominal pressure regulation, addressing trunk function impairments associated with brainstem-related respiratory dysregulation [16,17]. Prior to intervention, the child frequently demonstrated functional breath-holding during demanding motor tasks, likely as a compensatory strategy to increase trunk rigidity through elevated intra-abdominal pressure [33,34]. After 12 months of integrated therapy, this breath-holding behavior was observed less frequently, coinciding with apparent improvements in trunk stability and smoother transitional movements. This observation was based on repeated clinical assessment during functional tasks across multiple therapy sessions conducted over the intervention period. The reduction of the subcostal angle from 137° to 90° and the increase in sacral slope from 5° to 10°, both measured twice with a goniometer by an experienced pediatric physiotherapist, further suggest improved sagittal alignment and more efficient respiratory-postural coupling. Beyond ventilation, diaphragmatic activation contributes to intra-abdominal pressure modulation and provides proprioceptive input essential for trunk stabilization via phrenic afferents and trunk mechanoreceptors [33,35,36]. The observed increase in expiratory pressure from 10 mmHg to 25 mmHg may indicate improved expiratory pressure generation capacity, consistent with enhanced respiratory-postural interaction.

These mechanistic considerations are supported by quantitative functional outcomes observed during the intervention period. A 40-point increase in GMFM-88 over 12 months indicates substantial longitudinal change in gross motor performance during the intervention period, particularly in domains related to axial control and functional motor capacity. This magnitude of change exceeds previously reported minimal clinically important differences for gross motor function measures [13,15] and may suggest potential clinical relevance. Improvements in PEDI domains further reflect gains in functional independence and participation [37]. Although spontaneous developmental progression cannot be excluded in a growing child, the temporal association between the revised intervention strategy and measurable improvements is consistent with a potential treatment-related contribution. Importantly, prior to the introduction of the revised rehabilitation protocol at 2.5 years of age, motor development had remained largely static for approximately 9 months despite continuous conventional therapy and structured home-based intervention, suggesting a deviation from expected spontaneous developmental progression. Notably, these changes occurred during a period of high neurodevelopmental plasticity, when targeted sensory-motor input may exert amplified influence on emerging postural networks [38,39].

The identification of early hip migration underscores the importance of integrating orthopedic surveillance within neurorehabilitation pathways [40,41]. Children with developmental central hypotonia are at increased risk of hip dysplasia, subluxation, and progressive instability due to insufficient muscular stabilization and impaired postural control [40]. Daily supported standing in abduction was implemented with the aim of promoting symmetrical loading and potentially reducing progressive femoral head displacement, highlighting the importance of coordinated neuromotor, respiratory, and musculoskeletal management in JS. This multidimensional approach reflects the interdependence of postural control, respiratory function, and skeletal alignment in children with cerebellar and brainstem dysfunction [35,42].

During the intervention period, careful attention was given to the child’s tolerance to sensory and motor stimulation, as appropriate task intensity and engagement are essential for effective motor learning and neuroplastic adaptation. Therapy sessions were continuously monitored for signs of fatigue, increased incoordination, or deterioration in movement quality, as excessive fatigue may negatively influence motor performance and reduce the efficiency of movement execution in neurological conditions [43,44]. If such changes were observed, the intervention was temporarily paused or terminated. This approach aimed to avoid excessive sensory load while maintaining conditions that may facilitate adaptive motor learning. Such regulation of therapy intensity may be particularly relevant in children with cerebellar and brainstem dysfunction, in whom impairments in sensory integration, timing, and motor coordination may increase vulnerability to overstimulation and reduce the efficiency of motor adaptation [45,46]. Therefore, the therapeutic strategy emphasised adaptive motor learning through controlled, goal-directed activity rather than repetitive passive stimulation, consistent with contemporary principles of experience-dependent neuroplasticity [43].

This report has several limitations. First, its single-case design limits generalizability, and spontaneous developmental progression cannot be fully excluded as a contributing factor to the observed functional gains. Second, outcome assessments were performed as part of routine clinical care and were not conducted under blinded conditions, which may introduce potential measurement bias and observer-related influence. Third, although standardized tools such as GMFM-88 and PEDI were used, some postural and respiratory parameters, including subcostal angle and sacral slope, were obtained using clinical goniometric assessment, which may be subject to measurement variability despite efforts to ensure consistency. In addition, expiratory pressure was estimated using a PARI PEP System-S device, which is primarily designed for airway clearance therapy rather than standardized physiological pressure measurement; therefore, this parameter should be interpreted as a clinical estimate rather than a validated respiratory outcome measure. Furthermore, functional visual outcomes were based on caregiver-reported assessment and structured clinical observation rather than instrument-based quantitative visual testing. Neurophysiological biomarkers were also not available to directly document central nervous system reorganization. Nevertheless, the structured longitudinal design and use of multidimensional functional outcome measures may provide clinically relevant insight into rehabilitation-associated functional change in Joubert syndrome.

## 7. Clinical Implications

This case supports the clinical relevance of mechanism-oriented neurorehabilitation targeting axial stabilization and respiratory-postural coordination in children with Joubert syndrome. Early implementation of interventions addressing trunk control, respiratory integration, and supported weight-bearing may facilitate functional progression and may help reduce secondary musculoskeletal complications. The findings also emphasize the importance of longitudinal monitoring using standardized outcome measures, as motor scales alone may not fully capture clinically meaningful changes. Multidimensional assessment integrating motor, respiratory, and postural domains may provide a more sensitive framework for evaluating rehabilitation outcomes in cerebellar and brainstem disorders. Further prospective studies are needed to establish standardized rehabilitation protocols and disease-specific functional outcome sets for Joubert syndrome.

## 8. Conclusions

This longitudinal case report describes functional changes observed during a structured 12-month neurorehabilitation programme in a child with Joubert syndrome. Improvements were observed across several outcome measures, including GMFM-88, PEDI, and selected postural–respiratory parameters, suggesting changes in gross motor performance, functional independence, and postural organization during the intervention period.

These observations highlight the potential value of structured longitudinal outcome monitoring in rare neurodevelopmental disorders and illustrate a possible rehabilitation approach targeting postural and respiratory–motor integration in Joubert syndrome. Further prospective studies using standardized multidimensional outcome measures are required to better characterize rehabilitation-associated functional trajectories in this population.

## Figures and Tables

**Table 1:** Summary of Diagnostic Findings.

Domain	Age	Key Findings	Clinical Interpretation	Functional Relevance
Prenatal ultrasound	22 weeks’ gestation	Cerebellar vermis dysgenesis and enlarged fourth ventricle	Suspicion of posterior fossa malformation requiring postnatal confirmation	Early indicator of disrupted cerebellar development affecting postural control maturation
Brain MRI	10 months	Vermian agenesis; thickened and elongated superior cerebellar peduncles; deepened interpeduncular fossa forming the molar tooth sign; small T2-weighted white matter hyperintensities	Pathognomonic neuroimaging features consistent with JS and cerebellar-brainstem malformation	Structural substrate underlying axial hypotonia, impaired postural control, and delayed motor development
Polysomnography	1 year	Sleep latency 112 min; AHI 5.4/h; OAI 1.7/h; CAI 1.7/h; arousal index 7.2/h; nadir SpO_2_ 77%	Moderate mixed sleep apnea with clinically significant oxygen desaturation	Brainstem-related respiratory dysregulation affecting autonomic stability and postural-respiratory integration
Otolaryngology evaluation	1 year	Adenoid hypertrophy (~75% nasopharyngeal obstruction); obstructive sleep apnea	Upper airway obstruction contributing to sleep-disordered breathing	Increased respiratory load potentially exacerbating compensatory trunk stiffening and motor inefficiency
Ophthalmologic assessment	1.5 years	Bilateral hyperopia and astigmatism documented on autorefractometry and cycloplegic refraction (OD approx. +3.75 to +4.00 D; OS approx. +3.00 to +4.75 D with astigmatic component); early gaze instability and rotatory nystagmus	Refractive error and oculomotor dysfunction consistent with cerebellar and brainstem involvement	Impaired visual stabilization affecting balance, spatial orientation, and motor coordination
Pelvic radiography	2.5 years	Asymmetric lateral migration of femoral heads with reduced acetabular coverage	Early hip displacement associated with axial hypotonia and impaired postural control	Secondary musculoskeletal consequence of reduced axial stability and delayed weight-bearing
Genetic testing (WES)	3 years 11 months	Two heterozygous variants in the *B9D2* gene: c.513C>G (exon 4) and c.146C>T (exon 3; variant of uncertain significance)	Variants associated with Joubert syndrome and related ciliopathies	Molecular support for the clinical and radiological diagnosis

Abbreviations: MRI, magnetic resonance imaging; T2, T2-weighted imaging; AHI, apnea-hypopnea index; OAI, obstructive apnea index; CAI, central apnea index; SpO_2_, peripheral oxygen saturation; OSAS, obstructive sleep apnea syndrome; ENT, ear, nose, and throat; PSG, polysomnography; OD, oculus dexter (right eye); OS, oculus sinister (left eye); D, diopters; WES, whole-exome sequencing.

**Table 2:** Multidimensional functional outcomes before and after 12 months of neurorehabilitation.

Domain	Measure	Baseline	12 Months	Absolute Change	Relative Change (%)
Gross motor function (GMFM-88)	Total score (% of maximum)	12	52	+40	+333%
	A. Lying and Rolling (%)	42	96	+54	+129%
	B. Sitting (%)	15	85	+70	+467%
	C. Crawling and Kneeling (%)	5	72	+67	+1340%
	D. Standing (%)	0	18	+18	NA
	E. Walking (%)	0	0	0	0
Functional independence (PEDI)	Mobility (Scaled Score)	8	40	+32	+400%
	Self-care (Scaled Score)	15	45	+30	+200%
	Social function (Scaled Score)	25	50	+25	+100%
Ataxia severity	modified BARS (0–30)	22	15	−7	−32%
Respiratory-postural alignment	Subcostal angle (°)	137	90	−47	−34%
Pelvic alignment	Sacral slope (°)	5	10	+5	+100%
Respiratory function	Expiratory pressure (mmHg, PARI PEP S level 1.5)	10	25	+15	+150%

Abbreviations: GMFM-88, Gross Motor Function Measure-88; PEDI, Pediatric Evaluation of Disability Inventory; mBARS, modified Brief Ataxia Rating Scale (range 0–30); PEP, positive expiratory pressure; mmHg, millimeters of mercury; %, percent; °, degrees. Interpretation: Changes are presented as baseline and follow-up values together with absolute and relative change. Absolute change is expressed in percentage points for GMFM-88, scaled score points for PEDI, mBARS points for ataxia severity, degrees for angular measures, and mmHg for expiratory pressure. Relative change (%) was calculated as (follow-up − baseline)/baseline × 100. Relative change was not calculated when baseline values were zero. Positive values indicate improvement, except for mBARS and subcostal angle, where lower values reflect reduced impairment and improved postural-respiratory organization. Measurement note: Expiratory pressure was measured using a PARI PEP S device connected to a manometer at a standardized resistance level (1.5), with the child positioned supine to ensure measurement consistency and minimize compensatory trunk activation.

**Table 3:** Longitudinal caregiver-reported functional visual outcomes between 1 and 3.5 years of age.

Domain	Functional Parameter	Age 1 Year	Age 3.5 Years	Change	Clinical Interpretation
Visual attention	Sustained visual fixation	Reduced	Improved	Positive	Improved visual engagement
Visual tracking	Ability to follow moving objects	Impaired	Improved	Positive	Improved visuomotor integration
Visual responsiveness	Reaction to visual stimuli	Reduced	Improved	Positive	Improved sensory responsiveness
Visual field use	Exploration of environment	Limited	Improved	Positive	Improved spatial awareness
Visuomotor coordination	Reaching toward visual targets	Impaired	Improved	Positive	Improved sensorimotor coordination
Object recognition	Recognition of familiar objects	Limited	Present	Positive	Improved visual cognition
Visual exploration	Active visual scanning	Reduced	Increased	Positive	Improved environmental interaction
Multisensory integration	Integration of vision with movement	Impaired	Improved	Positive	Improved functional visual use
Functional visual behavior	Interaction with environment using vision	Limited	Present	Positive	Improved adaptive function

## Data Availability

Data supporting the findings of this study are available from the corresponding authors upon reasonable request.

## Abbreviations

The following abbreviations are used in this manuscript:

JS	Joubert syndrome
MRI	Magnetic resonance imaging
GMFM-88	Gross Motor Function Measure-88
PEDI	Pediatric Evaluation of Disability Inventory
mBARS	Modified Brief Ataxia Rating Scale
WeeFIM	Wee Functional Independence Measure
DNS	Dynamic Neuromuscular Stabilization
Vineland-II	Vineland Adaptive Behavior Scales, Second Edition
SpO_2_	Peripheral oxygen saturation
AHI	Apnea-hypopnea index
OAI	Obstructive apnea index
CAI	Central apnea index
PSG	Polysomnography
PEP	Positive expiratory pressure
mmHg	Millimeters of mercury
OD	Oculus dexter
OS	Oculus sinister
D	Diopters
AFOs	Ankle-foot orthoses
AAC	Augmentative and alternative communication
WES	Whole-exome sequencing
